# How hatred of abortion providers is propagated in social media: an investigation of YouTube videos

**DOI:** 10.1080/26410397.2025.2569200

**Published:** 2025-10-03

**Authors:** Paula Tavrow, Jenny Lee, Frankie Guevara, Ashley Lopez, Cate Schroeder, Aparna Sridhar

**Affiliations:** a Adjunct Professor, Fielding School of Public Health, University of California, Los Angeles, CA, USA.; b MPH student, Fielding School of Public Health, University of California, Los Angeles, CA, USA; c MPH student, Fielding School of Public Health, University of California, Los Angeles, CA, USA; d MPH student, Fielding School of Public Health, University of California, Los Angeles, CA, USA; e Undergraduate student, University of California, Los Angeles, CA, USA; f Clinical Professor, Obstetrics and Gynaecology, UCLA Health, Los Angeles, CA, USA

**Keywords:** abortion providers, attacks, YouTube, social media, hate speech, mis/disinformation, *Dobbs* decision

## Abstract

Since abortion was legalised in 1973, the United States anti-abortion movement has sought to eliminate abortion services. One strategy has been to foment hatred of abortion providers, which legitimises anti-abortion activists’ attacks on providers and facilities, thereby dissuading pregnant people from seeking abortions and hindering providers’ willingness to offer services. After the 2022 *Dobbs* decision overturning *Roe v. Wade*, these attacks escalated. The goal of our study was to examine a social media platform, YouTube, to identify the categories of videos promulgated by the anti-abortion movement and to investigate how these videos might be propagating hatred of providers. We also sought to discern differences post-*Dobbs*. Using three search terms – “pro-life,” “abortionist” and “abortion providers” – we developed a sample of 291 YouTube videos with high viewership, of which 217 had content about providers or patients. Videos took numerous forms, including debates, testimonials and undercover investigations. We identified four major dimensions of abortion provider depictions, in order of frequency: manipulative (deceptive, greedy and biased), villainous (brutal murderers), uncaring (callously harming women) and immoral. Abortion facilities were characterised as “death camps” and abortions as “baby funerals.” Patients were reviled if they “celebrated” their abortions, but not if they were remorseful. Videos post-*Dobbs* seemed more geared to reducing demand by emphasising patient regret and provider harms. We concluded that despite YouTube content moderation, abortion providers were being maligned in videos, which potentially contributes to clinic attacks. To increase support for abortion providers, content creators may want to specifically extol providers’ contributions to public well-being.

## Plain language summary

Since abortion was legalised in 1973 through *Roe v. Wade,* the United States anti-abortion movement has tried to halt abortion services. One strategy has been to demonise abortion doctors. This could act as a deterrent for pregnant people, medical students, and current abortion doctors. Attacks on abortion doctors and clinics have increased since the 2022 Supreme Court decision overturned the right to abortion in the US. We examined videos created by the anti-abortion movement on a social media platform, YouTube. We wanted to identify different types of videos and to assess how these videos might be contributing to the hatred of abortion doctors. We were also interested in learning if there were any changes to the types of videos after the 2022 decision. Our final sample was 291 YouTube videos, of which 217 specifically discussed doctors or patients. Videos took many forms, including debates, patient and professional testimonials, and undercover investigations. We found that abortion providers were being portrayed in anti-abortion videos in four major ways. These were: (1) manipulative (deceptive, greedy and biased), (2) villainous (brutal murderers), (3) uncaring (harming women) and (4) immoral. Abortion facilities were called “death camps” and abortions “baby funerals.” Patients were mocked by anti-abortion groups if they “celebrated” their abortions, but not if they were regretful afterwards. Our findings suggest that videos after the 2022 decision aimed to reduce demand by emphasising patient regret and how doctors were hurting pregnant people. We concluded that anti-abortion videos on YouTube did indeed contribute to hatred of abortion doctors. This could have many negative consequences.

## Résumé

Depuis la légalisation de l’avortement en 1973 aux États-Unis, le mouvement anti-avortement dans ce pays a cherché à éliminer les services d’avortement. Une stratégie a consisté à attiser la haine envers les prestataires réalisant des interruptions de grossesse, ce qui a légitimé les attaques des militants opposés à l’avortement contre les prestataires et les installations, dissuadant ainsi les personnes enceintes d’avorter et entravant la volonté des prestataires de proposer leurs services. Après l’arrêt Dobbs qui a annulé Roe v. Wade, ces attaques ont redoublé d’intensité. Notre étude visait à examiner une plateforme de médias sociaux, YouTube, pour identifier les catégories de vidéos diffusées par le mouvement anti-avortement et analyser comment ces films peuvent propager la haine envers les prestataires. Nous souhaitions aussi identifier les différences après Dobbs. À l’aide de trois termes de recherche – « pro-vie », « avorteur » et « prestataires d’avortement » – nous avons constitué un échantillon de 291 vidéos à forte audience sur YouTube, dont 217 concernaient des prestataires ou des patientes. Les vidéos prenaient diverses formes, notamment des débats, des témoignages et des enquêtes en caméra cachée. Nous avons identifié quatre dimensions principales dans la représentation des prestataires d’avortement, par ordre de fréquence: manipulateurs (trompeurs, cupides et partiaux), infâmes (meurtriers brutaux), indifférents (nuisant sans scrupules aux femmes) et immoraux. Les centres d’avortement étaient qualifiés de « camps de la mort » et les avortements de « funérailles de bébés ». Les patientes étaient injuriées si elles « célébraient » leur avortement, mais non si elles éprouvaient des remords. Les vidéos après Dobbs semblaient davantage axées sur la réduction de la demande, en mettant l’accent sur les regrets des patientes et les préjudices causés aux prestataires. Nous en avons conclu qu’en dépit de la modération des contenus de YouTube, les prestataires d’avortement étaient calomniés dans les vidéos, ce qui contribue potentiellement aux attaques contre les cliniques. Pour accroître le soutien aux prestataires d’avortement, les créateurs de contenu peuvent vouloir vanter spécifiquement les contributions que font ces agents au bien-être public.

## Resumen

Desde la legalización del aborto en 1973, el movimiento antiaborto en Estados Unidos ha buscado eliminar los servicios de aborto. Una estrategia ha sido fomentar el odio hacia prestadores de servicios de aborto, lo que legitima los ataques de activistas antiaborto contra los prestadores de servicios y establecimientos de salud y, por ende, disuade a las personas embarazadas de buscar abortos y obstaculiza la disposición de los prestadores de servicios a ofrecer servicios. Después que la decisión del caso *Dobbs* de 2022 revocó *Roe c. Wade*, los ataques se intensificaron. El objetivo de nuestro estudio era examinar una plataforma de las redes sociales, YouTube, para identificar las categorías de videos promulgados por el movimiento antiaborto e investigar cómo estos videos podrían estar propagando el odio hacia los prestadores de servicios. También pretendíamos discernir las diferencias post-*Dobbs*. Utilizando tres términos de búsqueda – “provida”, “abortista” y “prestadores de servicios de aborto” – creamos una muestra de 291 videos de YouTube con una alta audiencia, de los cuales 217 tenían contenido sobre prestadores de servicios o pacientes. Los videos tomaron diferentes formas, tales como debates, testimonios e investigaciones encubiertas. Identificamos cuatro principales dimensiones de representaciones de prestadores de servicios de aborto, en orden de frecuencia: manipuladores (engañosos, codiciosos y prejuiciosos), malvados (asesinos brutales), indiferentes (causan daños a las mujeres cruelmente) e inmorales. Las clínicas de aborto eran caracterizadas como “campos de muerte” y los abortos como “funerales de bebés”. Las pacientes eran vilipendiadas si “celebraban” su aborto, pero no si estaban arrepentidas. Los videos post-*Dobbs* parecían estar más orientados a reducir la demanda haciendo hincapié en el remordimiento de las pacientes y en los daños causados por prestadores de servicios. Concluimos que, a pesar de la moderación del contenido en YouTube, los prestadores de servicios de aborto estaban siendo difamados en los videos, lo cual posiblemente contribuye a los ataques contra las clínicas. Para aumentar el apoyo a los prestadores de servicios de aborto, los creadores de contenido deberían ensalzar las contribuciones de los prestadores de servicios al bienestar público.

## Introduction

Prior to 1973, only a few states in the United States (US) provided abortions as part of health care.^1^ Abortion access changed dramatically with the Supreme Court’s decision in *Roe v. Wade*, which determined that pregnant people had a constitutional right to abortion. In 1982, there were 2,908 facilities nationwide providing abortions.^2^ However, by 2017, this number had shrunk to 1,587 facilities, a decline of 45%.^2^ By the time of the *Dobbs v. Jackson Women’s Health Organization* decision in June 2022, overturning *Roe v. Wade*, abortions were accessible in only 11% of counties, and just 24% of obstetricians/gynaecologists provided abortion care.^3^

One reason for the decline in abortion access was the rise of harassment, intimidation and violence against abortion clinics and providers.^4–6^ Within a few years of *Roe v. Wade*, abortion clinics began experiencing bombings and arson, intimidation of patients, and direct attacks and threats on healthcare providers who offer abortion services (hereinafter referred to as abortion providers).^4,7^ In 1984, anti-abortion violence had reached “epidemic” proportions. Researchers noted that it was “the first time in our nation’s history that healthcare providers have been singled out as targets of violence in pursuit of a social agenda.”^1^ A study in 1985 found that 47% of abortion providers had experienced anti-abortion harassment.^4,8^ Attacks on abortion providers and attempts to stop them from offering abortion services have continued unabated over the years. On the eve of the *Dobbs* decision, 35% of 321 providers in one study reported harassment.^3^

Rather than take a victory lap, the anti-abortion movement seems to have been energised post-*Dobbs* to become even more violent against clinics.^5^ Since 1977, there have been 11 murders, 42 bombings, 200 arsons, 531 assaults, 492 clinic invasions, 375 burglaries, and thousands of other incidents of criminal activities directed at patients, providers, and volunteers.^9^ In the six months after the overturning of *Roe v. Wade*, the National Abortion Federation (NAF) reported that that violence grew exponentially: clinic invasions with burglary increased by 100% (from 5 in 2021 to 10 in 2022), bomb threats rose by 133% (from 3 to 7), clinic obstructions increased by 538% (from 45 to 287), and stalking of providers rose by 913% (from 8 to 81).^9^ Similarly, in their most recent National Clinic Violence Survey, the Feminist Majority Foundation found that 2022 was a particularly dangerous year for abortion providers, with the second-highest number of violent reports in over two decades.^10^ Abortion providers and staff have been targeted with threatening “Wanted” or “Killers Among Us” posters, along with the disclosure of private information on social media (e.g. photographs, addresses, places of worship and family member information).^10^

**Figure 1.**
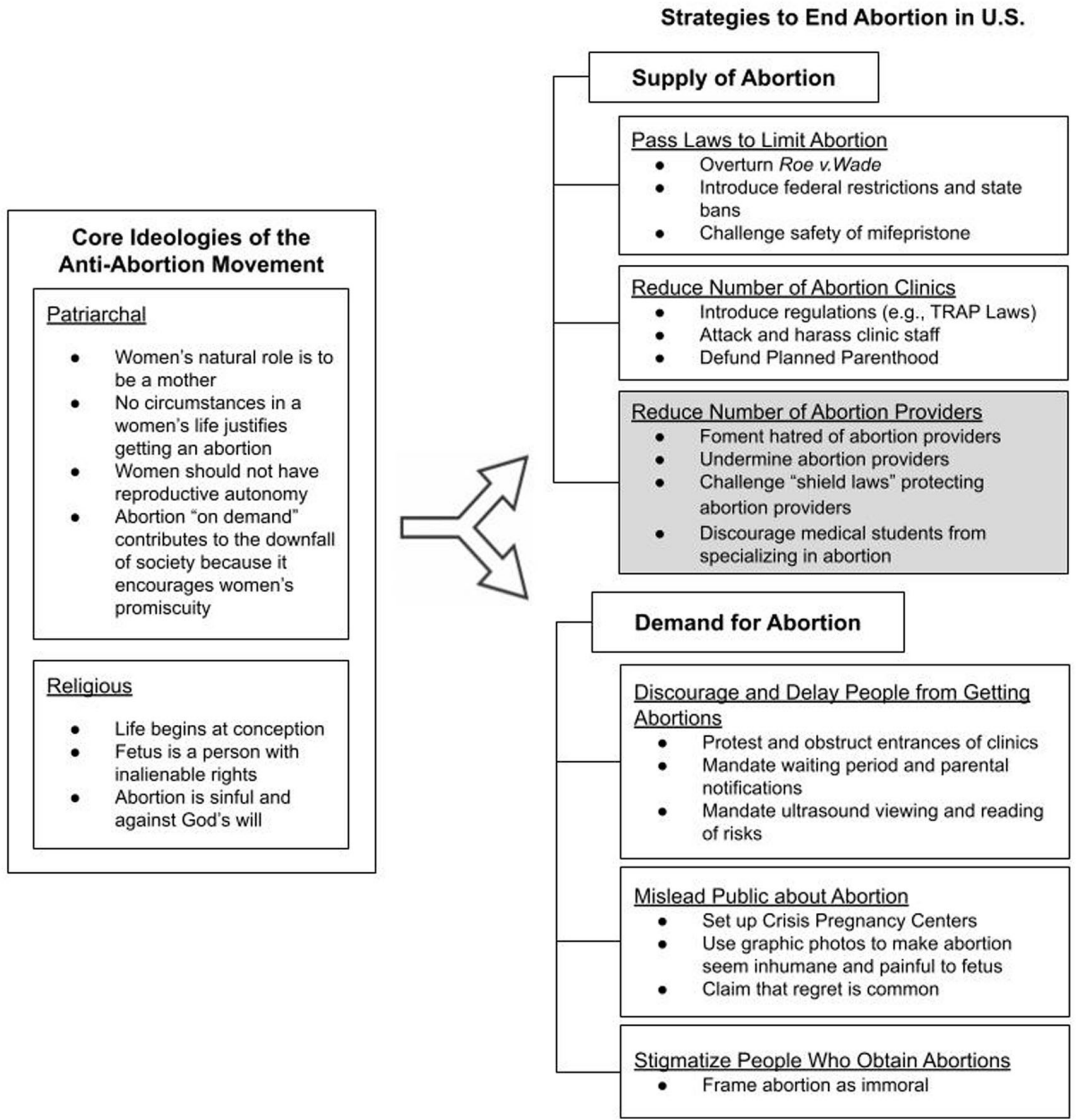
Core anti-abortion ideologies and strategies to end abortion in the United States

Attacking abortion providers – through physical violence, intimidation and general misrepresentation of what they do – has been a core strategy of the anti-abortion movement to reduce the supply of and demand for abortions in the U.S. (see Figure 1).^7^ Anti-abortion ideology is rooted in patriarchal and religious precepts that deny sexual and reproductive autonomy to women.* Traditional gender roles are romanticised, with women expected to fulfil their “natural role” by becoming mothers.^11^ Women who have abortions are deviating from this narrow path. Fundamentalist religions promote the notion that life begins at conception and fetuses should have the legal rights of persons. All pregnancies are seen as intentional gifts from God. Any attempt to alter this perceived natural course of life through abortion is viewed as sinful because it goes against God’s plan.^12^ The anti-abortion movement has worked relentlessly to end all abortions by creating legal and socioeconomic obstacles to deter not only pregnant people from obtaining abortions but also providers from performing them.

To decrease the supply of abortion, the anti-abortion movement appears to have focused on three main areas: (1) passing laws to limit abortions, (2) reducing the number of abortion clinics and (3) reducing the number of abortion providers. The cornerstone of this effort was to change the legal landscape of abortion by overturning *Roe v. Wade*. The Federalist Society, a conservative legal advocacy organisation created in response to *Roe*, has been influential in advising Republican presidents on the selection of conservative, anti-abortion candidates for federal and Supreme Court nominations.^13,14^ Conservative legislators restricted abortion access through the passage of the Hyde Amendment in 1976, which prohibited the use of any federal funds for abortion. Since the *Dobbs* decision, they have sought to limit the use of mifepristone, a core component of medical abortion.^15^ To reduce the number of clinics offering abortion services, these legislators also passed numerous “TRAP” (Targeted Regulation of Abortion Providers) laws, which imposed onerous requirements on abortion providers.^6^ They have also sought repeatedly to defund Planned Parenthood, which offers abortions as well as other reproductive health services.

To reduce the number of abortion providers, the movement has sought to foment general hatred of abortion providers as well as malign them among their peers and ancillary staff. This may discourage medical students from either entering the profession or choosing to learn how to perform abortions (including miscarriage management).^16^ In states with post-*Dobbs* abortion bans, abortion training may be difficult to access, thereby diminishing over time the number of doctors with abortion skills.^17^ Some of these states have also been challenging “shield laws,” which protect abortion providers in non-banned states who offer telehealth or mail abortion medications across state lines.^18,19^ Within the medical community, abortion providers experience a lack of support from their peers, resulting in restrictions on the scope of practice, remuneration, and professional development.^16,20^ Abortion providers report constantly having to defend the legitimacy of their practice, citing examples of other physicians not wanting to share waiting rooms or staff unwilling to assist with scheduling.^20^ While some abortion providers report finding the situation amusing, others find it highly exasperating.^21^ Abortion providers are both deeply respected by some for their expertise and simultaneously ostracised by others because of it.^21^ They have also been shut out by institutions or face discrimination by credentialing committees due to their specialty, all of which exacerbate feelings of isolation and their ability to practice.^20^ Internally, abortion providers face “moral injury” – a feeling of conflict between knowing what is right for their patients and facing legal repercussions (e.g. criminal prosecution, incarceration) as a result of acting on it.^22^ These experiences leave many abortion providers feeling frustrated, stressed and burnt out, which causes some to discontinue their practice.^23^

The anti-abortion movement has also engaged in strategies to decrease demand for abortions. A key strategy has been to delay people from obtaining abortions through picketing clinics and obstructing entrances.^5^ Anti-abortion state legislators have also implemented mandatory waiting periods and parental notification for minors, which can make it more difficult to obtain abortions within state-mandated gestational age limits. Requirements for patients to view an ultrasound and providers to read a list of risks are further intended to dissuade pregnant people from moving forward with an abortion. Another strategy has been to mislead the public about various aspects of abortion, such as safety and efficacy, or whether fetuses suffer pain. One approach has been to set up Crisis Pregnancy Centres across the country, which entice pregnant people with free pregnancy testing, on-site ultrasounds and short-term assistance.^24^ These Centres, designed to resemble bona fide women’s health clinics, purvey disinformation about the short-term and long-term consequences of abortion, such as breast cancer, infertility, miscarriage and depression.^24^ Lastly, the movement seeks to heighten the stigma attached to abortion by making people feel ashamed and guilty for engaging in a purportedly immoral act.

Anti-abortion groups, such as the National Right to Life, use social media to strengthen networks among like-minded individuals and increase the circulation of anti-abortion content.^19^ Social media platforms enable organisations to find potential followers and allow individuals to see content tailored to their interests, reinforcing their beliefs. Direct messaging and public replies have created new, convenient ways to communicate hatred and promote violence.^25^ A recent example is the way the right-wing extremist organisation, Proud Boys, used Telegram to coordinate their violent activities.^26^

One of the most popular social media platforms is YouTube, launched in 2005 and now used by over 85% of American adults.^27,28^ About 64% of adult users have encountered videos that contain misinformation while using the site.^28,29^ As of December 2020, YouTube was estimated to have removed 73,000 videos that promoted violence, 259,000 harmful or abusive videos, and 1.4 million misleading videos in violation of community guidelines.^30^ Following the *Dobbs* decision, YouTube sought to address abortion misinformation on its site by introducing a policy to remove videos displaying “unsafe or alternative” abortion methods not validated by health authorities.^31,32^ YouTube also committed to attaching information panels to all abortion-related content and search results to provide viewers with credible information sources outside of its platform.^31^ While the platform declared its intention to address hate speech, its current guidelines do not include specific professions, such as abortion providers, in its protected categories.^33^

Despite YouTube’s 2022 post-*Dobbs* stated policy to remove videos with misleading information on abortion, we wished to assess whether the platform might still be hosting videos that incited hatred against abortion providers. Our main questions were:

- What types of YouTube videos do anti-abortion organisations or individuals use to sway public opinion against abortion providers and procedures?
- Within these videos, how are abortion providers and procedures depicted? Are those who obtain abortions also under attack?
- Have there been any notable changes since the *Dobbs* decision?

To date, there has not been any systematic investigation of how videos on a popular social media platform might be fomenting hatred of abortion providers. Based on the results of our study, we aimed to provide recommendations to YouTube and other social media sites on how they might reduce the perpetuation of hatred and adopt more comprehensive hate speech policies. Another goal was to inform pro-choice activists about how social media has been used to foster hatred of abortion providers, so as to enable more effective counter-programming.

## Methods

### Sample design and inclusion criteria

To obtain YouTube videos that were likely to align with the anti-abortion position, our study team used the search terms, “pro-life” and “abortionist.” We then added a third search term, “abortion provider,” which lacked the ideological connotations of the other terms, but could potentially yield content relevant to our inquiry. We also tested the term “abortion industry.” In a search of YouTube, we found that “abortion industry” yielded far fewer videos than the other terms, essentially duplicating videos found with “abortionist,” so we did not use it. We conducted the searches and compiled the videos in May 2023.

We next filtered each search term by a specific timeframe (e.g. 4–20 minutes) and sorted the videos by the number of views (i.e. most popular). Our aim was to analyse videos with high viewership. With three search terms and three timeframes (<4 minutes, 4–20 minutes, 21–45 minutes), we ended up with nine categories. We opted to exclude videos exceeding 45 minutes due to budget constraints and probable saturation (analysing several 60–90 minutes videos did not produce novel content). The team obtained the first 75 videos for each category after the filters were applied, because our goal was to include videos with 150,000 views or more. After about 75 videos, view counts generally fell to below 100,000. This approach generated 675 videos uploaded over a sixteen-year period by anti-abortion activist groups such as Live Action, right-wing news sites, and individuals with pseudonymous usernames.

Our third step was to delete 195 duplicates and 54 videos lacking transcripts. YouTube provides auto-generated transcripts of most videos, but sometimes transcripts are not available because the audio did not sync with the video or multiple people were talking over each other. We next removed 135 video games,^†^ videos not in English or from countries outside the US, or videos from “pro-choice” sources such as self-identified progressive comedians.

### Content analysis

The study team collectively viewed about 25 videos to develop initial thematic codes and standardise coding procedures using Quirkos, a qualitative data analysis software tool. The team began by generating codes for the YouTube-generated transcripts for providers and patients to describe any hateful discourse related to abortion providers. Examples of initial provider codes were “immoral” and “murderer”; examples of initial patient codes were “regret” and “coercion” (i.e. being pressured to get an abortion). The team then expanded the coding to include pro-choice activists and allies, to determine if they were being labelled and disparaged in the same way as providers. Due to limitations of the Quirkos software, it was not possible to code images.

Each coder individually coded about 45 videos. Because the study team had six coders who were undergraduate and graduate students, to improve consistency, the principal investigator and the coders met weekly to discuss coding, jointly review videos that were difficult to code, and add new codes that arose inductively, such as “callous” and “anti-science.” Each coder would first watch a video and then code the transcript, because sometimes the transcript was garbled and not understandable on its own. The coding entailed manifest content analysis to obtain counts of terms like “baby killer,” as well as latent content analysis to elucidate the various ways that providers’ purported brutality was manifested (without directly labelling it as such). Once the initial coding was completed, pairs of team members reviewed the data by code to assess the accuracy of the coding, resolve discrepancies and identify top quotes that best exemplified each code. Any unresolvable discrepant interpretations were brought back to the entire team for discussion.

After resolving any discrepancies, the team grouped the codes into four dimensions, which seemed to best encompass the codes. We next determined what were the main categories of videos. Then we mapped the four dimensions onto the most common video categories to assess the main sources for each dimension. We then sought to determine whether abortion patients also were being reviled. Lastly, we examined changes in rhetoric after the *Dobbs* decision, using 30 June 2022, as the cut-point between pre- and post-*Dobbs* videos in our sample. Four videos were uploaded between the *Dobbs* decision of 24 June 2022 and 30 June 2022. We placed these in the pre-*Dobbs* category because the content seemed to have been created before the *Dobbs* decision. All four were in different categories.

Regarding ethical considerations, the study analysed YouTube videos that had been posted for public viewing, had already been widely watched, and were not password-protected. In addition, no usernames of those who uploaded the videos or created the content are included in this paper. An exemption was granted by the University of California at Los Angeles Institutional Review Board (11 February 2023) because the study did not meet the definition of human subjects research. Informed consent was not required for the same reason.

### Positionality

Our approach to evaluating the hatred directed at abortion providers in YouTube videos uploaded by pro-life organisations is grounded in our backgrounds as public health scholars and practitioners at UCLA. As a team, we bring a deep commitment to reproductive justice, with expertise in healthcare, social science and reproductive rights. One of our authors is a practising abortion provider, which gives us insight into the personal and professional challenges faced by those who offer abortion care. We view abortion as an essential component of healthcare, central to reproductive autonomy and the broader goal of health equity. We are concerned that restrictions on abortion disproportionately harm people of colour, low-income individuals and those in rural areas.

We recognise that anti-abortion rhetoric is not only a matter of moral belief but is also tied to political, social, and cultural forces that perpetuate inequality. Our analysis considers the ways in which pro-life organisations use platforms like YouTube to spread hate, stigmatise abortion providers, and create hostile environments that threaten both individual safety and public health. We reject efforts to restrict reproductive rights or silence those who provide abortion care, seeing these actions as part of a larger movement to control individuals’ bodies and deny them their rights. At the same time, we acknowledge that individuals who hold anti-abortion views often do so from deeply held moral convictions, and we aim to engage in critical examination without demonising opposing beliefs. Ultimately, our work seeks to contribute to a respectful, evidence-based dialogue while defending the human rights and dignity of those who provide and seek abortion care.

## Results

Our final sample consisted of 291 YouTube videos uploaded between 22 November 2007 and 4 May 2023. Of these, 217 had content relevant to abortion providers or the procedure. Our findings are presented in two sections: (1) video categories and (2) content of videos. In the first section, we describe the various formats of videos used by anti-abortion groups and assess whether these formats seemed to change after the *Dobbs* decision. In the second section, we present the results of our content analysis of the video transcripts and discuss post-*Dobbs* changes. Because our methodology was skewed towards more popular YouTube videos, the average viewership per video category was generally about 1,000,000.

### Video categories

Of the 291 videos we viewed, 57% were uploaded prior to the *Dobbs* decision (including six days afterwards), and 43% were uploaded afterwards. We determined that the videos could be grouped into eight categories (see Table 1). No video was placed in more than one category. The overarching purpose of the anti-abortion videos we viewed was to convince people that abortion was unacceptable and harmful. The maligning of doctors who performed abortions seemed to be an integral part of many videos. Another purpose of the videos was to paint the pro-choice movement and individual abortion supporters as violent, illogical, hysterical, unable to sustain an argument and immoral. Some videos were clearly efforts to “flip the script” and show that everything of which the anti-abortion movement has been accused is actually a characteristic of the pro-choice movement.

**Table 1.** Categories of anti-abortion videos on YouTube, by timeframe (pre- or post-*Dobbs***)**

Video category	Pre-*Dobbs* [*n* = 166] (100%)	Post-*Dobbs* [*n* = 125] (100%)	All videos [*n* = 291] (100%)
**1. Debating logical fallacies of pro-choice**	**42** (**25.3%)**	**36** (**28.8%)**	**78** (**26.8%)**
Street “conversions,” in which anti-abortion advocates approach people on the street and attempt to change their minds.	13 (7.8%)	14 (11.2%)	27 (9.3%)
Anti-abortion people directly “schooling” pro-choice people, arguing with their beliefs, and pointing out why they are wrong.	15 (9.0%)	3 (2.4%)	18 (6.2%)
Videos designed to “debunk” classic pro-choice arguments.	8 (4.8%)	6 (4.8%)	14 (4.8%)
Kristan Hawkins “debating” an audience of university students.	5 (3.0%)	7 (5.6%)	12 (4.1%)
YouTubers reacting to videos of pro-choice advocates’ arguments and critiquing their logic/views.	1 (0.6%)	6 (4.8%)	7 (2.4%)
**2. Crusaders**	**29** (**17.5%)**	**37** (**29.6%)**	**66** (**22.7%)**
Lila Rose: founder and president of Live Action.	4 (2.4%)	25 (20.0%)	29 (10.0%)
Everyday advocates and protesters: ordinary people supporting the anti-abortion movement and speaking their opinions.	7 (4.2%)	7 (5.6%)	14 (4.8%)
Religious crusaders: clergy and other religiously motivated.	10 (6.0%)	3 (2.4%)	13 (4.5%)
Ben Shapiro: conservative political commentator and host.	8 (4.8%)	2 (1.6%)	10 (3.4%)
**3. Caught on camera**	**33** (**19.9%)**	**13** (**10.4%)**	**46** (**15.8%)**
Physical violence and verbal abuse by the pro-choice side.	13 (7.8%)	12 (9.6%)	25 (8.6%)
Undercover investigations (in-person or recorded phone calls).	20 (12.0%)	1 (0.8%)	21 (7.2%)
**4. Personal testimonials**	**13** (**7.8%)**	**21** (**16.8%)**	**34** (**11.7%)**
People who chose abortion and now regret it.	2 (1.2%)	16 (12.8%)	18 (6.2%)
People who chose *not* to have an abortion and stand by their decision	7 (4.2%)	4 (3.2%)	11 (3.8%)
People with disabilities or their families speaking about how abortion harms the disabled and/or advocating for anti-abortion policies.	4 (2.4%)	1 (0.8%)	5 (1.7%)
**5. Professional testimonials**	**18** (**10.8%)**	**3** (**2.4%)**	**21** (**7.2%)**
Former abortion providers or clinic staff.	16 (9.6%)	1 (0.8%)	17 (5.8%)
Physicians/scientists/professors with anti-abortion perspectives presenting their views or evidence to support the anti-abortion side.	2 (1.2%)	2 (1.6%)	4 (1.4%)
**6. Both Sides: “finding common ground” videos, neutral news segments on the two sides, and balanced debates.**	**13** (**7.8%)**	**5** (**4.0%)**	**18** (**6.2%)**
**7. Mocking pro-choice people**	**7** (**4.2%)**	**9** (**7.2%)**	**16** (**5.5%)**
Videos intended to deride or humiliate pro-choice people, by showing how disorganised or “chaotic” they are, such as showing them at a rally looking “dumb.”	6 (3.6%)	5 (4.0%)	11 (3.8%)
Satirical videos intended to ridicule pro-choice people and paint them in a negative light, without any direct “debating.”	1 (0.6%)	4 (3.2%)	5 (1.7%)
**8. Graphic Imagery: videos using visuals to convey the “brutality” of abortion procedures.**	**11** (**6.6%)**	**1** (**0.8%)**	**12** (**4.1%)**

We labelled the most common category as “debating logical fallacies of pro-choice” (27%). These videos gave anti-abortion proponents an opportunity to mock pro-choice activists, some of whom seemingly changed their minds instantly when presented with information on the brutal “realities” of the abortion procedure or of providers’ motives. Other videos in this category showed pro-choice people being schooled on their supposed illogical notions, sometimes in “debates” on university campuses. The next most common were videos we called “crusaders” (23%). These consisted of charismatic religious or conservative commentators, clergy or activists using moral suasion and hyperbolic terms to motivate their followers or influence undecided people. The third category we labelled as “caught on camera” (16%) videos, which showed altercations with pro-choice activists or undercover investigations of abortion clinics.

The next two categories consisted of either personal (12%) or professional (7%) testimonials. The personal testimonials were from people who either regretted their abortion, did not have an abortion and stood by their decision, or might have been aborted due to disability. The personal testimonials generally conveyed emotional struggles, beliefs that abortion was immoral, concerns about long-term mental health consequences of abortion, feelings of failure or desperation and calls for action to restrict abortion access. Professional testimonials were mainly from former abortion practitioners, staff, or scientists who espoused anti-abortion sentiments and gave detailed comments on the procedure itself.

The last three categories of videos consisted of what we called “both sides” (6%), which depicted neutrally the two major positions or tried to find common ground, “mocking pro-choice people” (6%), which used humiliation or satire, and “graphic imagery” (4%), which sought to portray the purported brutality of the abortion procedure.

In examining whether any shifts seemed to have occurred post-*Dobbs*, the “crusader” and “personal testimonials” video categories appeared to have increased. Meanwhile, “professional testimonials” and “caught on camera” appeared to have decreased, possibly because overturning *Roe* made these less necessar*y*. The “graphic imagery” category also seemed to decline.

### Content of videos

**Figure 2.**
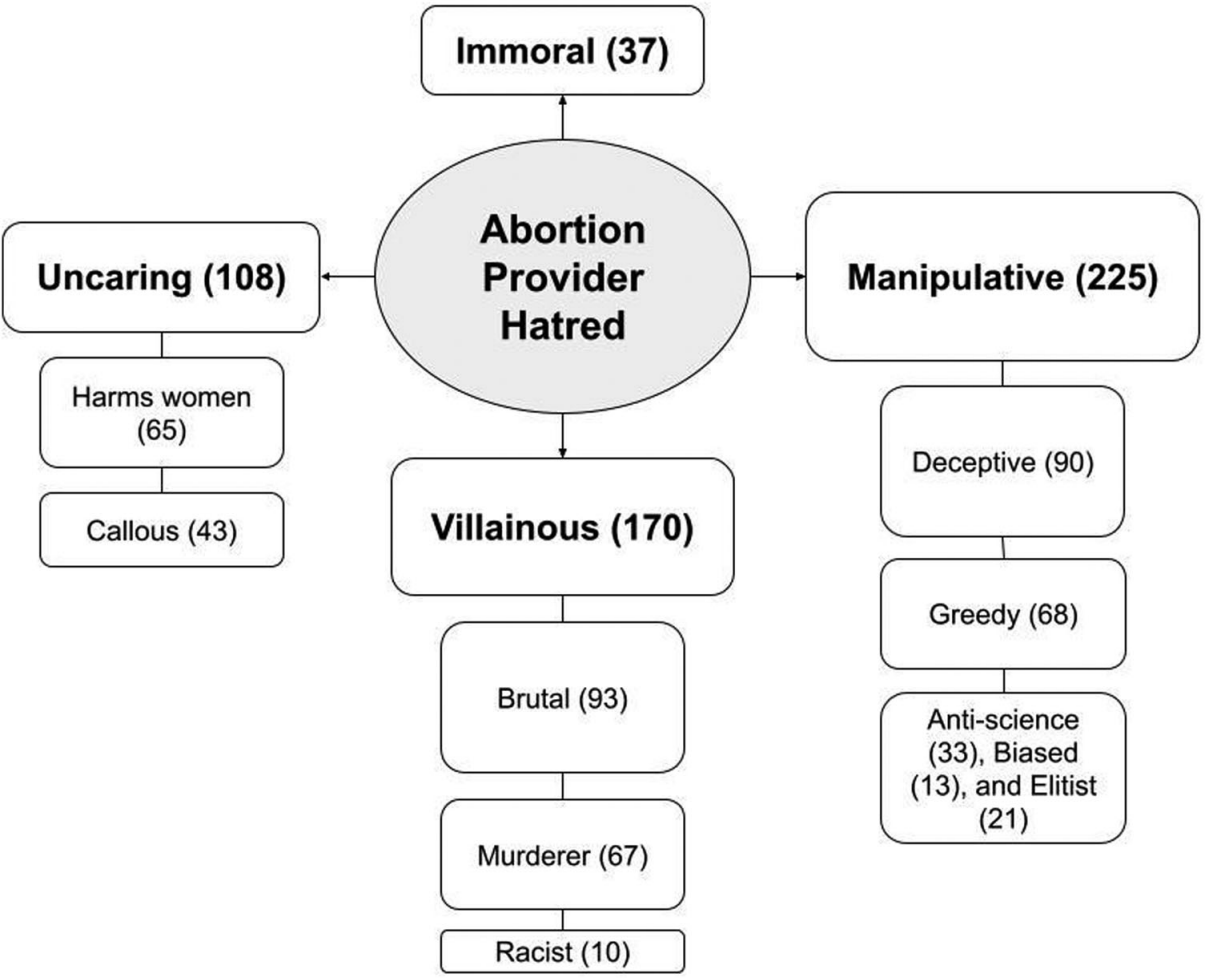
Main dimensions and sub-dimensions describing abortion providers found in YouTube anti-abortion videos, by the number of instances

In analysing the content of the YouTube videos, we found 518 unique instances where abortion providers or their procedures were vilified in some way. Most videos included more than one form of hatred. We categorised the hatred into four major dimensions: *manipulative, villainous, uncaring,* and *immoral.* Each dimension except immoral had 2–4 sub-dimensions (see Figure 2). We did not find any positive depictions of abortion providers or the procedure in our sample of anti-abortion videos. We found that patients were mainly pitied, but were scorned if they “celebrated” their terminations. After the *Dobbs* decision, we discovered fewer depictions of providers as manipulative and more emphasis on their supposed villainy and callousness.

#### Manipulative

The most common characterisation of abortion providers was manipulative. About 42% of the transcripts concerning providers contained passages depicting them as having ulterior, nefarious motives, thus creating a gulf between them and other doctors.

### Deceptive

Allegations that abortion providers deceive clients constituted the largest sub-dimension in this category. In some videos, former patients claimed that abortion providers hid their true intentions. They accused abortion providers of attempting to gain their trust and dissuade them from the “natural” path of motherhood, by validating their desires to terminate a pregnancy to continue school or pursue a career. Former patients complained that abortion providers deceptively framed abortion as a simple solution to their problems, rather than informing them that it would be a life-altering negative experience.

“*… we have been sold the story that the most innocent members of our society are stealing our freedom. Mothers everywhere have been told to turn on the children growing within their wombs and have been guided to take their lives.*” [215, pre-Dobbs]

Anti-abortion videos blamed abortion providers for withholding key information from patients, so that patients would think that abortion was their only option. Abortion providers were accused of giving “misinformation” about the risks of abortion or the extent of fetal anomalies, which meant that the women’s “so-called right to choose in most cases is not a choice at all.” In personal testimonials, former patients stated that abortion providers would attempt to mislead them about abortion by speaking in euphemisms.

“*They've used words that are meant to make us feel more comfortable when talking about taking the life of an innocent child in its mother's womb. They've coddled us into hearing the word ‘abortion’ and not really listening to what it means.*” [215, pre-Dobbs]

“*What about calling that baby ‘a clump of cells’? I think that's harmful misinformation because then you're encouraging people to kill it like it's nothing, when it's actually a human life.*” [228, post-Dobbs]

Providers’ supposed deception extended into contraception. For example, in one video, a former abortion provider declared that her clinic encouraged teenage girls to have sex to increase the number of abortions the clinic performed. She described dispensing low-dose birth control pills, which are efficacious only if ingested at the same time every day, knowing that teenagers would have difficulty following this regimen and thus would get pregnant. She also claimed that her clinic would purchase “cheap condoms” that broke easily, thereby causing unintended pregnancies and increasing the demand for abortions. Other videos insinuated that abortion providers promote sexual promiscuity and deceive young people about contraceptive methods to keep themselves in business.

### Greedy

Another major sub-dimension was that abortion providers acted selfishly for financial gain. Instead of referring to abortion as a part of healthcare, videos described the procedure as the “highest revenue-generating product,” stating that abortion providers “sell” abortions to patients instead of trying to prevent them from occurring. Former abortion clinic staff claimed that abortion providers prioritised making money over a patient’s best interest, calling into question their professional integrity.

“*All seven of those [abortion] doctors falsified the records … They worked very hard to learn how to get Medicaid funds and they wanted to increase their tax funding to seven hundred million a year … It was a profit center and we were well on the way to opening more clinics … They [doctors] didn't care if the instruments weren't clean … They just wanted to get their money and get through the day and go*.” [701, pre-Dobbs]

Anti-abortion videos branded Planned Parenthood as the face of the “abortion industry.” Despite its nonprofit status and the range of services it offers, Planned Parenthood clinics were portrayed as businesses that sought to profit off pregnant women by convincing them to have abortions. Several videos claimed that clinics must meet their abortion “quotas,” which implies that they need a certain number of abortions to stay afloat. Other videos purportedly “exposed” abortion clinics and providers as profit maximisers who sought to make money from selling aborted fetuses or tricking people into “buying” abortions.

### Biased, elitist, and anti-science

Anti-abortion videos frequently depicted abortion providers as defaulting to recommending abortions when a fetal abnormality was detected. One of the core beliefs of the anti-abortion movement is that a fetus should have the same rights as a person, which means that any suggestion to end a pregnancy is a threat to human life. Therefore, abortion providers were accused of being ableist when making a medical recommendation to end a pregnancy, even if the fetus had a lethal condition or would have an exceedingly poor quality of life. Former patients claimed that they felt pressured into choosing an abortion because the doctor was considered the expert. They portrayed the provider as an elitist who “knows better than them,” which left patients feeling inferior and distressed. Some former patients even accused providers of “falsely diagnosing” fetal anomalies to convince pregnant women to end pregnancies.

“*[The NICU team] said, we need to tell you what might happen today. When your baby's born at 29 weeks, here's all the things: blindness, brain bleed, bones that aren't formed correctly … When she was born by emergency C-section, she didn't have any abnormalities. So, everything in their laundry list of things that could happen, none of it happened*.” [155, pre-Dobbs]

“*… [abortion providers have] an extreme extraordinary position that's built on this idea of dehumanizing the child in the womb, which is what the abortion position is saying: they're not human. If they're conceived in violence, if they have a disability, if they're really sick, kill them*.” [404, post-Dobbs]

Anti-abortion videos also claimed that abortion providers do not correctly interpret science due to their bias towards abortion, relying on personal values instead of “scientific facts” when making their recommendations. In these videos, viewers were encouraged to question whether abortion providers are equipped to make medically and scientifically sound decisions relative to life and death. Because abortion providers do not subscribe to the anti-abortion movement’s definition of personhood, some videos insinuated that they were not credible and were against science.

“*It is a scientific certainty that life begins at fertilization. At the moment a sperm fuses with an egg (known as fertilization or conception), a new, unique human being that is genetically distinct from both parents comes into existence*.” [544, pre-Dobbs]

“*Abortion is never medically necessary. Thousands of medical experts agree, despite the lies coming from abortion industry and abortion allies in this country. The intentional direct killing of a baby is never medically necessary*.” [414, post-Dobbs]

#### Villainous

The second most common dimension, found in about 30% of the transcripts, portrayed providers as “murderers” or criminals. These videos used brutal and graphic language to describe abortion procedures as babies being “tortured” and then “executed.” These videos sought to instil guilt and shame not only among potential patients, but also their partners and family members. Labelling abortion providers as “baby killers” maligned them in the medical community as well. Several anti-abortion videos accused providers of wishing to “suck [babies] out of existence,” particularly of Black women, who are disproportionately more likely to seek abortions. Instead of depicting abortion doctors as providing a desired service to people with unwanted pregnancies, videos portrayed them as villains who do not mind inflicting “pain” on the “unborn.”

### Brutal

In this category, the most common sub-dimension concerned the purported brutality of abortion providers. Many videos characterised terminations as grossly inhumane. Dilation and evacuation were described as “you grab a limb, you twist and you pull” or “crushing of the skull, the sucking out of the brain.” A few videos sought to depict medical abortion as equally brutal, such as “[using] pills to starve the baby of nutrients” or “delivering a dead baby in a toilet.” The vast majority of abortions discussed were later in pregnancy, when the procedure might involve keeping track of what is extracted to prevent subsequent infection from any retained parts. Because most viewers are not familiar with standard medical practice, videos discussing “stacking up body parts on the side of the table” could seem grotesque. Anti-abortion videos never acknowledged that most abortions occurred in the first trimester, when a fetus was barely discernible.

“*If you crush down on the instrument, white material runs out of the cervix. That was the baby's brains and you could pull out skull pieces … Sometimes a little face comes back and stares back at you*.” [601, pre-Dobbs]

“*Abortions use suction or forceps to violently dismember a child while he or she is still alive, followed by crushing the chest cavity or head of that baby. Or the abortionist uses large needles to inject poison into the child’s head, or into her heart, or they exsanguinate the child, meaning they drain the blood from the baby's body or the abortionist uses pills to starve the baby of nutrients until he or she dies*.” [405, post-Dobbs]

Several widely-viewed videos showed former abortion providers explaining how they became disillusioned with the work due to its brutality. They stated that the procedure made them “physically sick,” which led them to exit the profession. Former clinic staff recalled that fetal remains were “just thrown into the dumpster” or “put in a Pyrex dish in the freezer.” Others commented on how abortion was a form of barbarism, “like torturing a puppy in your backyard … ”. Inflicting “immense pain” on a fetus was declared unjustifiable for any reason, but especially if the pregnancy was mistimed. Anti-abortion videos juxtaposed a woman’s “comfort” with the “violence” experienced by the fetus to foster guilt for being “selfish.” Abortion providers were portrayed as not just complicit in this torturing of “innocent life,” but as the instigators.

“*… a baby will be injected with a substance that causes cardiac arrest as the mother is induced to deliver her stillborn baby, so the [mother] simply isn't inconvenien[ced]. Human lives are not inconvenient*.” [215, pre-Dobbs]

### Murderer

Describing an abortion provider as a “murderer” was the next largest sub-dimension in this category. Because the anti-abortion movement believes in the personhood of a fetus, abortion was portrayed as an intentional mechanism to “kill babies.” Some anti-abortion videos claimed that providers took advantage of the power dynamic between physician and patient to achieve the killing of a “weaker human being”. Indeed, a number of videos likened abortion to past genocides, and individual providers to well-known murderers. The most frequently referenced historical event was the Holocaust. The “dead babies” that resulted from abortion were equated to children who perished in death camps like Auschwitz. Terms such as “execution,” “dismemberment” and “slaughter” were all used to describe abortion providers’ day-to-day work.

“***Narrator:** Two years later after reading an article comparing abortion with the Nazi Holocaust, [the provider] saw herself as a mass murderer. **Former provider:** I probably murdered more people than Ted Bundy or any of the mass murderers if you consider all the abortions that I did*.” [604, pre-Dobbs]

“*It’s like [abortion providers are] almost hunting [babies] down and saying ‘we're going to execute you'.*” [215, pre-Dobbs]

Another narrative focused on fetal anomalies. Some videos questioned why providers do not let fetuses with life-threatening conditions “die naturally” rather than “use tools to kill them.” They noted that doctors do not kill people in a coma, and wondered why fetuses were not accorded the same rights. A termination was labelled as a “cold and violent death” at the hands of an “abortionist.” Several videos lamented that abortion providers were killing so many babies that there were none left to adopt.

### Racist

The last sub-dimension was that abortion providers had a racist agenda. While uncommon, some videos highlighted historical injustices to portray providers as motivated to cleanse the country of Black and brown people through eugenics. The main approach adopted was to distort the current mission of Planned Parenthood, claiming the past ideologies of Margaret Sanger were still “wove[n] into the fabric” of the organisation. The videos sought to associate increased access to reproductive health care with heightened predation on people of colour.

“*Planned Parenthood, they are coming with mobile abortion clinics … driving close to the borders of the red states [and] trying to lure pregnant women to these clinics. Right, these people must really really love killing Black babies, because they're all Black*.” [232, post-Dobbs]

“*We understand the history of abortion in that Margaret Sanger, the founder of Planned Parenthood, brought abortion to the Black communities because we were growing at alarming rates and Hispanic communities were growing at alarming rates. They brought abortion to our community to ensure we didn’t procreate at the rates we were*.” [440, post-Dobbs]

#### Uncaring

The third dimension of abortion providers as uncaring was found in nearly 20% of the transcripts about providers. Former patients frequently accused providers of being indifferent or callous to women’s suffering, both physical and mental, which caused even more pain. Indeed, former patients cited providers’ perceived coldness and lack of sympathy as some of the reasons that they became uncomfortable with their decision to abort. When the whole process felt transactional, some patients stated that they regretted their decision to terminate.

### Harms women

More than half of the videos in this category discussed how abortion providers were so uncaring that they casually inflicted physical and mental harm on patients and failed to warn women of potential long-term risks. The aftermath of the procedure was consistently painted as bleak and negative. Some anti-abortion videos claimed that abortions could result in infertility, breast cancer, perforation of the uterus, collapse of fallopian tubes or negative mental health outcomes (e.g. depression, suicide), with women resorting to alcohol and drugs as coping mechanisms. Former patients who felt guilt or regret declared that providers had misled them into believing that abortions would result in their “problems be[ing] solved” or would “secure [their] future.” According to personal testimonial videos, providers were making unfulfilled promises to women whose lives did not improve after the abortion. Several videos also claimed that providers tell rape survivors that having an abortion would help them heal, when “[in reality] abortion doesn’t erase the assault” and instead causes “additional trauma.” No videos mentioned the potential harm to women of giving birth instead of having an abortion.

“*It’s the poor people who are preyed upon to have abortions, and it’s the poor people who then spend years struggling with post-abortion syndrome as well*.” [203, pre-Dobbs]

“*… when something is ripped from you, it leaves a hole. A big ugly hole. I never imagined what was going to happen physically, mentally, emotionally, spiritually. What I know now is that, whatever decision somebody chooses, is forever*.” [438, post-Dobbs]

“*Abortion did not help me. It did not secure my future, it did not make my future better, it hurt me, and it hurt my future. It actually stole and robbed a precious human being that should have been a part of my life and that's something that I can never change or get back*.” [413, post-Dobbs]

### Callous

Another sub-dimension was that providers were callous and devoid of emotion, rather than compassionate and empathetic. Several former providers claimed they became insensitive and callous as coping mechanisms. Former patients and staff complained that providers were short with their explanations and provided little support to patients seeking reassurance or emotional validation. One video declared that abortion providers saw fetuses as a “cancer” to be removed. Several patients described feeling like they were in a “drive-through,” where providers were more focused on speed and efficiency than on the person getting the abortion. A few patients reported informing their doctor of pain and not being given a caring response. If any complications arose, abortion providers were accused of not taking responsibility or following-up with the patient, but instead advising them to consult their local emergency department. Some videos expressed annoyance that abortion providers were not more emotional since their work involved implementing “the death penalty” for fetuses.

“*… when it was all ready to go, the [abortion] doctor came in. If [the patient] was lucky, he might make eye contact with her, might even say her name, usually not, and they would do the abortion and they would leave*.” [710, pre-Dobbs]

#### Immoral

The last dimension identified concerned the supposed immorality of abortion providers. Many anti-abortion videos described the “natural order of life” in which a woman is destined to be a mother, and giving birth is part of “God’s plan.” Since abortion care inherently went against this natural order, abortion providers were labelled as “evil.” Some videos depicted abortion providers as an extension of “Satan,” who tricked women into believing that abortion was safe and harmless. Pregnant women were counselled to resist providers’ “temptations.” A patient’s desire to pursue a career or continue their education was minimised by anti-abortion videos as choosing fleeting pleasures at the expense of their “unborn child.” Because children were considered “miracles of God,” providing abortions was considered sinful and hurt society.

“*… [as an abortion provider] you have to break down the natural modesty [of girls]. You have to become the expert in their lives and you have to separate them from their parents and their values. How do you do all of that? Well, sex education sells abortions*.” [716, pre-Dobbs]

To bring home the idea that abortion was immoral, some videos compared it to slavery. Anti-abortion videos framed their activism to ban abortion as a modern-day abolition movement. They argued that they cannot stand by as “people kill innocent children,” just as abolitionists could not just watch as African-Americans were enslaved and dehumanised. Videos stated that, similarly to enslaved people, pro-choice individuals and abortion providers denigrated the fetus. Anti-abortion groups, in contrast, maintained that the fetus has the same value as a “living and breathing human being” and should be rescued or set free.

“*If you love your neighbour as yourself, you don’t watch as they are being violently dismembered, torn limb from limb in pain. If you love your neighbour as yourself, you don’t watch from the doorstep as they are being dragged away to death, you rescue them from being taken away to slaughter. You hold them back from the slaughter*.” [461, post-Dobbs]

### Mapping of dimensions onto video categories

**Figure 3.**
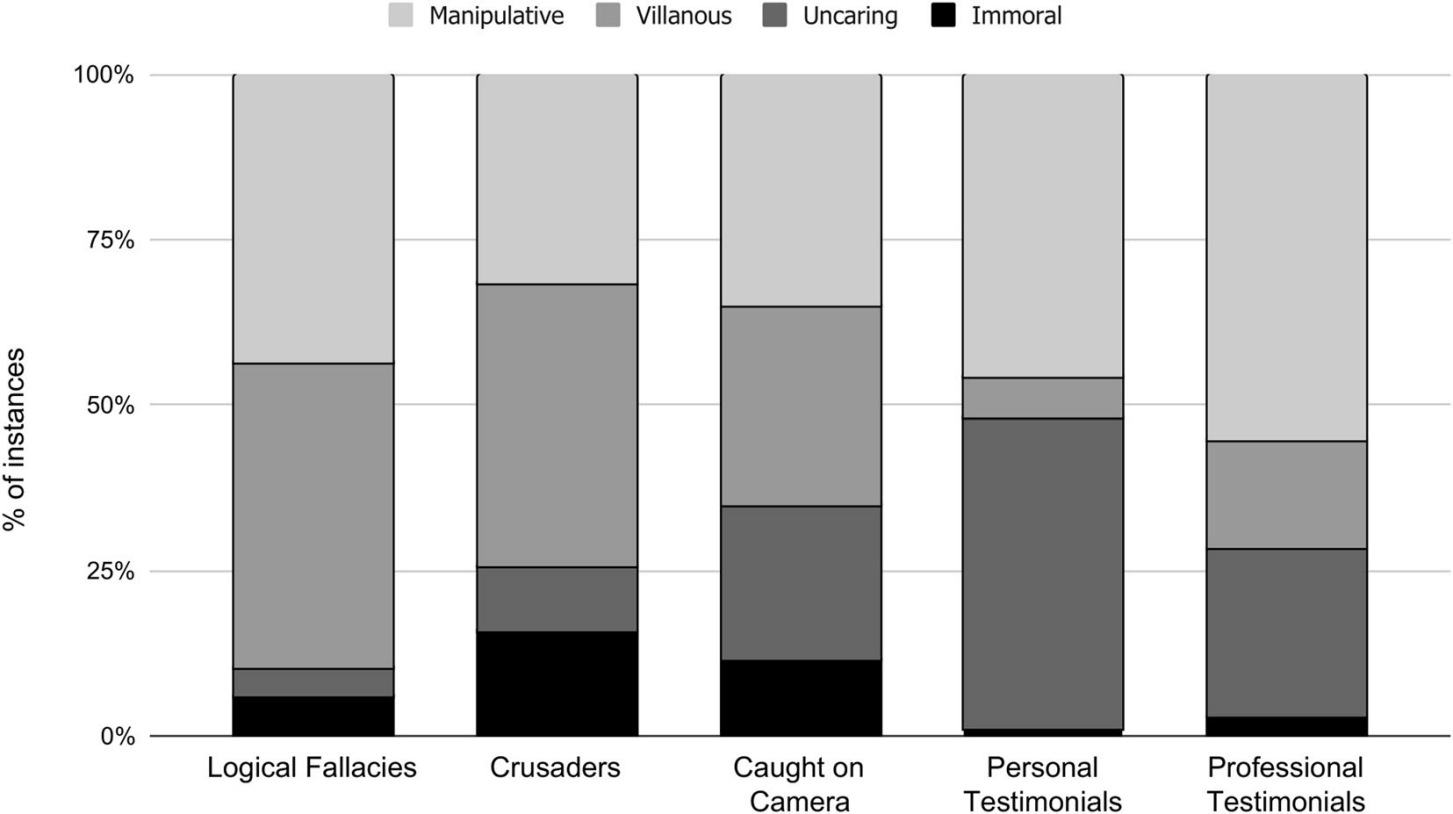
Proportion of negative provider characteristics found in the top five anti-abortion YouTube video categories

After identifying the main dimensions, we mapped them onto the five most common anti-abortion video categories, which together constituted about 84% of the videos viewed (see Figure 3). We found that portraying providers as manipulative was a core component of professional and personal testimonials, but it was also prominent in the other video categories. Focusing on providers’ purported villainy was central to videos featuring crusaders and debaters, but almost never occurred in personal testimonials. In contrast, personal testimonials frequently depicted providers as uncaring, which also figured into some professional testimonials and “caught on camera” videos. Lastly, providers’ supposed immorality was most commonly found in crusaders’ and “caught on camera” videos, but was almost never discussed in the testimonials.

### Characterisation of patients

In addition to abortion providers, we examined how abortion patients were depicted in the anti-abortion videos. Unlike providers, we found that patients were not direct targets of hate. The anti-abortion movement seemed aware that hostility towards pregnant women was not advantageous. Some videos explicitly stated that patients should not be prosecuted, because it would further victimise them, especially if their pregnancy was a result of sexual violence. Abortion patients who later showed remorse for their decision were met with acceptance and forgiveness, in part to gain additional voices for the anti-abortion cause.

“*There’s no major pro-life voice in America who advocates for prosecuting the mother … number one because it is counter-productive … Your goal is to convince women that they shouldn’t abort their babies, not to threaten them with punishment. You want them to make the moral choice … Basically honey is going to win people over more than vinegar*.” [640, post-Dobbs]

“*We’re not just trying to convince somebody not to make this decision [to have an abortion], but after a wrong decision is made, we’re going to combat this and we're going to help you correct this decision*.” [115, post-Dobbs]

One notable exception was pregnant people who “celebrated” their abortions and shared their relief from an unwanted pregnancy. These former patients challenged the anti-abortion narrative that abortion led to negative consequences. Some anti-abortion videos directly attacked these people as immoral and unwilling to repent for their “sin,” thus unworthy of forgiveness.

“*Her natural response is to celebrate, presumably both the casual sex and then also the abortion. How does this make anybody a better human being? How does this make society better? How does this make femininity better? How does this strengthen womanhood in any real way? … If your freedom is the killing of an unborn human being and celebrating it and fist-pumping it while you do it, making fun of people who are depressed and upset about doing it … what utter…*” [658, pre-Dobbs]

### Before and after the *Dobbs* decision

**Figure 4.**
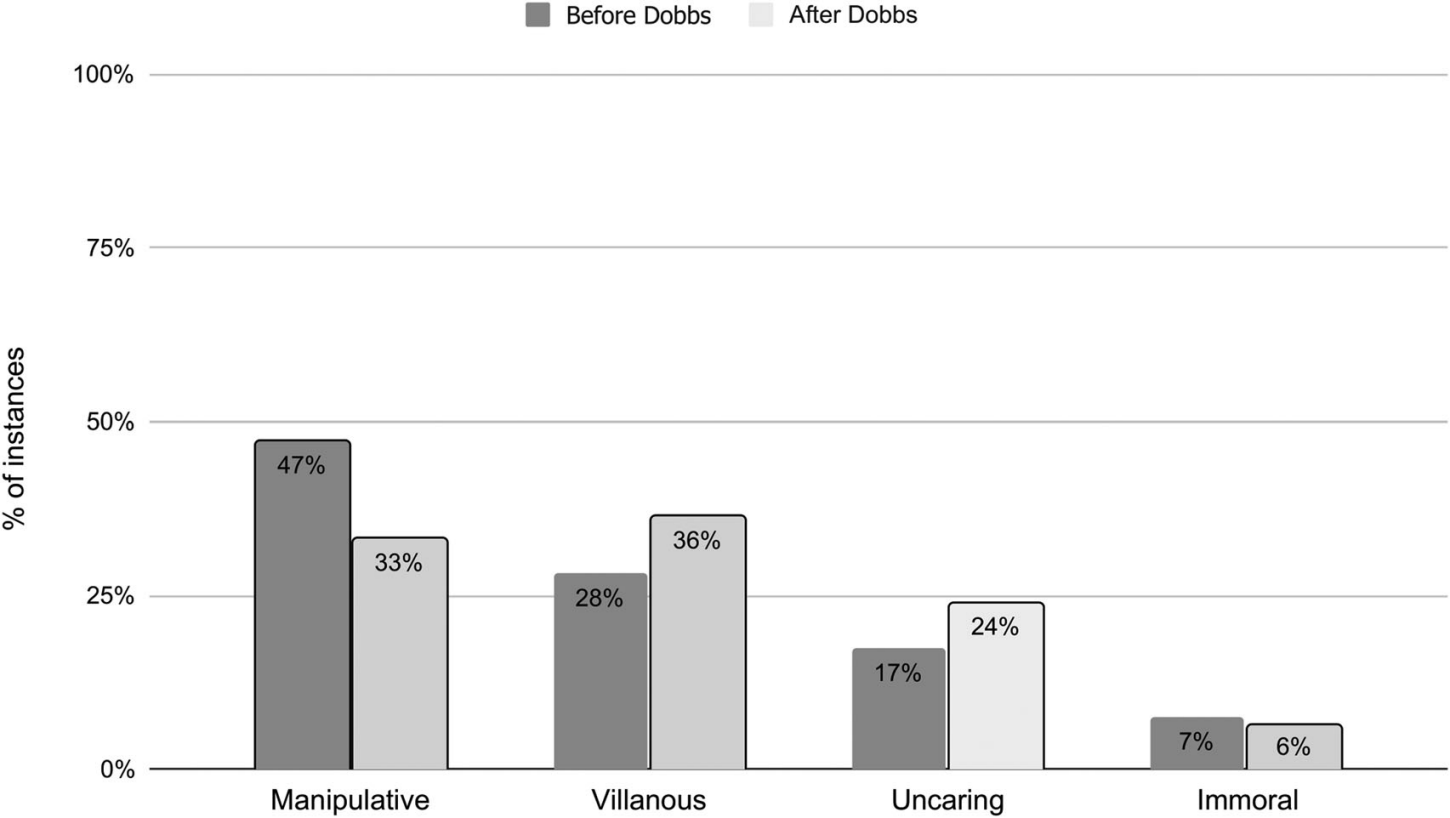
Negative abortion provider characteristics found in anti-abortion YouTube videos, before and after the *Dobbs* decision

To evaluate the possible effect of the *Dobbs* decision on how anti-abortion videos characterised providers, we analysed the frequency of dimensions in the two time periods (see Figure 4). Prior to the *Dobbs* decision, almost half of the YouTube videos we analysed depicted abortion providers as manipulative. Following the decision, the proportion of videos focused on providers’ deceit decreased, while being villainous and uncaring increased. Providers’ supposed immorality was essentially unchanged. Overall, anti-abortion videos shifted post-*Dobbs* from portraying providers largely as devious/transactional to describing them as brutal and callous.

## Discussion

The anti-abortion movement has spent decades seeking to end abortion in the United States. Part of that campaign has been to impugn the integrity, motives and morality of abortion providers on social media like YouTube. By depicting abortion providers as evil and callous, the movement has tried to lend credence to the argument that abortion was wrong and should be banned. Portraying providers as manipulative, deceitful and villainous was also a strategy to dissuade pregnant people from seeking an abortion, and to discourage medical students from entering a seemingly disreputable profession. When the anti-abortion movement characterises providers as biased towards abortion due to greed or ableist beliefs, people might feel uneasy about following their medical advice. No videos clarified that patients have the final say in whether to have an abortion.

### Hyperbolic language

The anti-abortion videos we viewed were permeated with overheated rhetoric, likening abortion providers to serial killers or executioners, abortion clinics to death camps and fetuses to enslaved people in need of rescue. Vilifying abortion providers as “conducting genocide,” “being proponents of child sacrifice” and “taking a vulnerable life despite detection of a heartbeat” was used to create a false power dynamic, with the provider as an abuser inflicting pain and harm on victimised pregnant people and fetuses. Labelling obstetrician/gynaecologists who perform abortions as “abortionists” was a way to reduce their expertise to a single “business” activity and to insinuate that many people needed to “buy” terminations for the providers’ work to be lucrative.

One of our main findings was the constant use of graphic and violent language to describe an abortion procedure, which was intended to elicit feelings of disgust and revulsion towards abortion providers and intensify stigma. In the literature, “abortion stigma” is the disgrace and shame of an individual who has either received or is associated with (e.g. supporter, abortion provider) an abortion.^34^ Anti-abortion groups believe that abortion providers are engaged in “dirty work,” which should discredit them in all aspects of their lives.^34,35^ Because mainstream healthcare generally has not included abortion provision, many abortion clinics are freestanding, which can heighten the public perception that abortion providers are engaged in secret, illicit activity.^36^ Researchers have found that providers performing second or third-trimester abortions are more likely to experience stigma.^36^ The most severe consequences of abortion stigma and hatred are overt discrimination, harassment and acts of violence against abortion providers.^34^

Most abortions occur in the first trimester, yet anti-abortion proponents portrayed all surgical abortions as much later (21 weeks or more of gestation). Inflammatory testimonials from former abortion providers recounted the “twisting of arms and legs” and “white fluid leaking from cracked skulls” to perpetuate the myth that virtually all fetuses were fully formed when aborted. In actuality, only one percent of abortion procedures are later in pregnancy, meaning it would be rare for abortion providers to encounter developed ligaments and organs during a surgical procedure.^37^ However, the increasing use of medical abortion in the first trimester removed one of the weapons from the anti-abortion arsenal, since it was more difficult to portray the ingestion of mifepristone and misoprostol pills as a brutal ordeal.

Anti-abortion videos often tried to co-opt language related to personal choice and civil rights. Some videos argued for the “choice of the girl in the womb,” reinforcing fetal personhood by equating fetal rights to prevent being aborted with reproductive rights. Other videos claimed to want to help sexual assault survivors, by maintaining that abortion should not be promoted as a panacea and would instead subject survivors to additional trauma. Yet if survivors stated that their healing depended on having an abortion, some in the anti-abortion movement would not allow them to have that choice. Elsewhere, abortion opponents have attempted to “flip the script” by claiming that perpetrators were protected when a fetus was aborted due to destroyed evidence.^38^ To gain favour from less conservative individuals, the anti-abortion movement has sought to portray itself as “pro-woman.” Using “mother–child” framing, anti-abortion proponents have sought to depict their purpose as being protectors of a vulnerable child.^38^

In our study, we found some anti-abortion rhetoric harkening back to historical grievances related to eugenics and efforts to reduce the fertility of “undesirable” people. For instance, a few videos claimed that Planned Parenthood was visiting Black neighbourhoods to lure people of colour into obtaining abortions. These videos labelling abortion providers as racists were relatively rare, possibly because they could redirect attention from abortion to systemic racism. While purported racism may not be a major dimension in social media, the anti-abortion movement did create the Life Education and Resource Network, whose explicit goal is to galvanise the Black community against Planned Parenthood by using genocidal arguments.^39^ Overall, anti-abortion activists have sought to manipulate and control the narrative through exaggerated language, disinformation, and co-opting of popular social justice movements.

### Providers cannot “win”

The anti-abortion videos we viewed put abortion providers in an untenable position. Testimonials from former patients who regretted their abortions accused providers of being transactional and callous. But doctors, not just abortion providers, often have full schedules and limited time for each patient, particularly in overburdened clinics. Some patients recalled meeting the abortion provider for the first time during the procedure and not being formally introduced. Others complained of no eye contact. We speculate that providers were aiming for a neutral, professional manner so that patients did not feel shamed or judged. This may have come across as cold. However, when providers made an effort to be caring and friendly, some former patients characterised them as being manipulative and biased towards abortion.

A similar paradox was uncovered when providers gave medical advice in cases of severe pregnancy complications or fetal anomalies. Protection of the patient’s health and negative fetal diagnoses are valid grounds for abortion recommendations, but were portrayed as coercive tactics and not scientifically sound. If the patient went ahead with the pregnancy and the baby was born without significant problems, this was portrayed as evidence that providers had a pro-abortion agenda, rather than an acknowledgment that it is impossible to predict birth outcomes with perfect accuracy. Former patients claimed that they had no say in the matter of choosing an abortion, even though they were the ultimate decision-makers. Interestingly, some patients complained of a power dynamic, with the provider having an unfair advantage due to their education and stature. One study found that portraying abortion providers as “predatory and exploitative” led to the enactment of state legislation that reinforced negative stereotypes, such as Tennessee’s “Freedom from Coercion Act,” which mandated that medical facilities display language emphasising patient autonomy and consent, implying that abortion providers were unduly coercive.^40^ On the other hand, when providers gave various options to patients and expressed uncertainty about the best choice, they were criticised for not giving a clear recommendation.

In sum, abortion providers were unable to satisfy anti-abortion critics. Those who established a personal connection with patients were viewed as manipulative, yet those who did not make the effort to do so were labelled as unsympathetic. Meanwhile, those providers who made medical recommendations for abortions were accused of not allowing patient input or respecting life, while those who encouraged patients to make their own decisions were criticised for not doing their job.

### Shifting anti-abortion strategies

The anti-abortion landscape has changed since the overturning of *Roe v. Wade*. In our review of anti-abortion videos, we noted a clear shift from focusing on the supply of abortion to seeking to reduce people’s demand for abortions. Prior to *Dobbs,* numerous videos fixated on “exposing” the brutality in abortion clinics and the deviousness of providers, mainly through testimonials of former abortion doctors and “caught on camera” investigations. Anti-abortion “crusaders” focused on convincing the public of providers’ greed, nefarious motives and immorality. Additionally, abortion practitioners were accused of misleading young women by advocating for comprehensive sexual education or providing contraceptives. Contraceptive use was portrayed as an intermediate step between “innocence” (sexual abstinence) and “sin” (having an abortion).

A year after the *Dobbs* decision, 13 states (Alabama, Arkansas, Idaho, Kentucky, Louisiana, Mississippi, Missouri, North Dakota, Oklahoma, South Dakota, Tennessee, Texas and West Virginia) immediately banned abortion or imposed severe limitations on gestational age, thereby restricting supply significantly in the US.^41^ In contrast, 17 states and the District of Columbia passed laws to protect the right to abortion (e.g. enshrined abortion into state constitutions or shielded abortion providers from prosecution).^41^ With abortion still available in many states, notable anti-abortion “crusaders” and former patients seemed to have renewed their efforts to convince the public that abortion irreparably harms pregnant people and is cruel to the fetus. Influencers released curated content depicting them as “successfully” countering pro-choice individuals’ logical “fallacies.” From patient testimonials, there appeared to be an increase in patients expressing profound regret for having had an abortion, and finding providers complicit because of their callousness. While we posit that anti-abortion content creators altered their emphases, we do not know whether YouTube changed its algorithms post-*Dobbs*, thereby affecting which videos were widely seen. There does seem to have been a flurry of anti-abortion videos with high viewership post-*Dobbs*, probably because of heightened interest in the subject due to the overturning of *Roe* and subsequent state legislative debates about the procedure.

Overall, post-*Dobbs* videos continued their inflammatory descriptions of abortion, emphasising long-term physical and psychological harm (e.g. infertility, severe depression), but seemed to opt for more “pro-woman” formulations to counter the “war on women” rhetoric from the left. While in general, those who underwent abortions were not vilified, we did find attention starting to be placed on individuals who celebrated their abortions. Because abortion stigma had not silenced these people, anti-abortion videos sought to shame and ridicule them in order to discourage others with positive abortion experiences from coming forward. By denying abortion opponents the narrative they have meticulously created that abortions harm women, those who describe positive experiences have become a new target of hatred.

### Limitations

Our study had several limitations. First, because content analysis is subject to interpretation, having six coders can decrease standardisation. However, reviewing all codes by pairs of team members and flagging issues to be discussed by the entire team reduced bias that might have been introduced by a single coder. A second limitation was that YouTube videos violating guidelines may have been removed before the research team could view them, particularly after the *Dobbs* decision, when YouTube announced its policy to remove abortion misinformation. It is possible that social media platforms with less content moderation would have more dangerous content and disinformation from providers. Third, some of our criteria for the removal of videos might have affected the findings. Not including videos longer than 45 minutes may have impacted the type and quantity of content we extracted. However, in general, more popular videos seemed to be shorter in duration, and our goal was to evaluate videos in wide circulation. The sample also included only videos with YouTube-generated transcripts. If videos with more graphic imagery lacked transcripts, our sample might have missed some violent content. Lastly, our keywords to search for videos may have affected the sample. If we had used more offensive terms for abortion providers, our sample might have included more extreme content. Nevertheless, we believe employing mainstream terms like “pro-life” and “abortionist” yielded videos that the general public would more likely view.

### Implications

This study found that anti-abortion videos on YouTube frequently maligned and misrepresented the work of abortion providers, even after heightened content moderation from YouTube following the *Dobbs* decision. YouTube’s 2022 moderation policy mostly seems to have involved affixing to all abortion-related videos a box containing “Abortion Health Information” from the National Library of Medicine. YouTube attests that it “doesn't allow content that poses a serious risk of egregious harm by spreading medical misinformation that contradicts local health authorities’ (LHAs) or the World Health Organization’s (WHO) guidance”, but none of the subsequent examples concerned abortion.^33^ In early 2025, YouTube quietly relaxed some of its content moderation policies and began allowing more violations of its rules on misinformation.^42^ However, it did affirm that, “Our goal remains the same: to protect free expression on YouTube while mitigating egregious harm.”^42^

While it is possible that some videos claiming abortion caused physical harm or promoting “abortion reversal” ((the misguided idea that medication abortions can be halted midway^43^) may have been removed under YouTube’s 2022 medical misinformation policy, other disinformation – particularly about the motives, deceit and callousness of abortion providers – proliferated on the platform.^30^ When it came to fetal anomalies, we found that anti-abortion videos on YouTube sought to entrench abortion providers as deceitful, ableist and biased, rather than as physicians interpreting symptoms and giving sound medical advice. This suggests that YouTube should consider expanding its hate speech policy to include certain occupations as a protected group status.^44^ Not only was YouTube’s policy ineffective in deterring certain types of abortion disinformation, but it also could be contributing to hatred of and attacks on abortion providers, which should be considered an “egregious harm.” Meanwhile, other social media platforms might wish to investigate how videos on their sites may be propagating hatred of abortion providers and improve their content moderation.

Another implication of the study is that some patient complaints could be mitigated, which might reduce hostility to providers. Some hatred undoubtedly would remain. But abortion clinics might wish to ramp up the aftercare they provide to patients, particularly psychological support services, to reduce abortion regret. Currently, most clinics do not offer post-abortion emotional care and only advise returning to the clinic for medical complications such as fever, heavy bleeding or intense cramping.^45^ Due to the large volume of patients seeking an abortion in states that still offer the services, abortion providers may not be able to provide the emotional support desired by some patients. However, more efforts could be made to help patients know the full range of options and to provide recommendations in cases of fetal anomalies without being too assertive. In addition, clinics could provide warm hand-offs to social workers or incorporate abortion doulas for patients who desire more extensive pre- or post-abortion counselling. Giving more attention to patients’ emotional needs might counter the complaint that providers were uncaring or that the abortion procedure was transactional.

Lastly, given the unending stream of hostility and disinformation directed at abortion providers from anti-abortion social media, hospital administrators and legislators need to be more proactive in ensuring that abortion providers feel valued and supported in the workplace. We recommend that professional colleagues and ancillary staff who engage in micro-aggressive behaviour or marginalise abortion providers be speedily reprimanded or penalised. We also urge pro-choice activists to produce videos that extol abortion providers’ important contributions to public well-being, highlighting their caring nature and destigmatising abortion procedures or medications. Those who had positive abortion experiences involving an abortion provider should also consider making a point of praising the provider.

## Conclusions

Social media has become the main source of news and information for many Americans. Researchers and policy-makers are increasingly interested in scrutinising social media to determine its potential effects on public attitudes. In this study, we sought to assess whether anti-abortion videos on one of the most popular platforms, YouTube, could be stigmatising and fomenting hatred of healthcare providers who offer abortions. We found that anti-abortion videos uploaded both before and after the *Dobbs* decision regularly depicted abortion providers as manipulative, villainous, uncaring and immoral. Some changes were noted after the *Dobbs* decision, but in general, the portrayals continued to be deleterious. With harassment and attacks on abortion clinics and providers on the rise after the *Dobbs* decision, it is imperative that YouTube, policy-makers and progressive activists take concrete measures to counteract these negative portrayals in order to reduce stress, burn-out and danger to abortion providers.

## Author contributions

*PT conceptualised the study, obtained funding, assisted with analysis of the data, drafted sections and edited the manuscript. JL conducted the literature review, drafted sections of the manuscript, developed figures, coded and analysed data. FG coordinated the study, developed the sample frame, coded and analysed data. AL coded and analysed data, reviewed codes for discrepancies. CS coded and analysed data, reviewed codes for discrepancies, and developed tables. AS conceptualised the study, gave insights into provider experiences, and commented on the manuscript. All authors reviewed and approved the final manuscript*.

## Footnotes

* While we recognise that not all pregnant individuals identify as women, the anti-abortion movement focuses strictly on people assigned female at birth and does not acknowledge transgender or non-binary pregnant people. Hence, whenever we discuss the anti-abortion movement’s viewpoint, we use their conceptualisation.

† The video games had “pro-life” or “abortion boy” in their titles but did not include abortion-related content.
